# Mentalization-Based Treatment

**DOI:** 10.1080/07351690.2013.835170

**Published:** 2013-11-15

**Authors:** Anthony Bateman, Peter Fonagy

**Affiliations:** Anthony Bateman, MA, FRCPsych is at Halliwick Personality Disorder Service, St Ann's Hospital, St Ann's Road, London, UK.; Peter Fonagy, Ph.D., FBA, is at Psychoanalysis Unit, University College London, Gower Street, London, UK.

## Abstract

The concept of mentalizing has captured the interest and imagination of an astonishing range of people—from psychoanalysts to neuroscientists, from child development researchers to geneticists, from existential philosophers to phenomenologists—all of whom seem to have found it useful. According to the Thompson Reuter maintained Web of Science, the use of the term in titles and abstracts of scientific papers increased from 10 to 2,750 between 1991 and 2011. Clinicians in particular have enthusiastically embraced the idea, and have put it to innovative use in their practices. Mentalization-based treatment (MBT)—making mentalizing a core focus of therapy—was initially developed for the treatment of borderline personality disorder (BPD) in routine clinical services delivered in group and individual modalities. Therapy with mentalizing as a central component is currently being developed for treatment of numerous groups, including people with antisocial personality disorder, substance abuse, eating disorders, and at-risk mothers with infants and children (A. Bateman & Fonagy, 2011). It is also being used with families and adolescents, in schools, and in managing social groups (Asen & Fonagy, 2011; Fonagy et al., 2009; Twemlow, Fonagy, & Sacco, 2005a, 2005b). In this article, we focus on MBT in the treatment of BPD.

## MENTALIZING AND BORDERLINE PERSONALITY DISORDER

Mentalizing is the process by which we make sense of each other and ourselves, implicitly and explicitly, in terms of subjective states and mental processes. It is a profoundly social phenomenon: as human beings, we generally (and automatically) form beliefs about the mental states of those with whom we interact, and our own mental states are strongly influenced by these beliefs. Nevertheless, human beings can temporarily lose awareness that others have minds, and can even at times treat one another as physical objects.

BPD is a complex and serious mental disorder characterized by a pervasive pattern of difficulties with emotion regulation, impulse control, and instability both in relationships and in self-image. BPD sufferers have a mortality rate, associated with suicide, that is 50 times that of the general population (Skodol et al., 2002). The dysfunction of self-regulation is particularly apparent in the context of social and interpersonal relationships (Posner et al., 2002) This may, to some extent, account for BPD patients’ generally poor response to traditional psychoanalytic treatment and their potential for symptomatic deterioration in the context of frequent treatment sessions (Stone, 1987).

MBT for BPD was founded on the specific theoretical basis that vulnerability to frequent loss of mentalizing is the underlying pathology that gives rise to these characteristic symptoms. In essence, we suggested that BPD was a disorder of mentalizing (A. W Bateman, 1998; Fonagy, 1998; Fonagy, Target, and Gergely, 2000). Our thesis was that patients with BPD temporarily and often lose the capacity to mentalize accurately within interpersonal interactions. This leaves them vulnerable to rapidly changing emotional states and impulsivity. In this article, we briefly cover some developmental aspects of mentalizing, discuss mentalizing's relationship to the treatment of BPD, outline the characteristics of the treatment method, provide an illustrative clinical example of a patient in individual therapy using MBT, and describe research on mentalization based approaches.

### Attachment, Mentalizing and BPD

Mentalization is not a static, unitary capacity, but a dynamic, multifaceted ability that has particular salience in the context of attachment relationships. Temporary lapses in mentalization are part and parcel of normal functioning, but the ability to continue to mentalize even under stressful circumstances, and a relatively fast recovery from lapses of mentalization, are the hallmark of robust mentalization. Robust mentalization is, in turn, strongly related to secure attachment (Fonagy et al., 2002). Secure attachment lays the groundwork for mature mentalizing later in life. Moreover, the ability to continue to mentalize even under considerable stress is associated with so-called “broaden and build” (Fredrickson, 2001, pp. 218–226) cycles of attachment security. These cycles reinforce feelings of secure attachment, personal agency, and affect regulation (*build*), and lead to development of more adaptive environments (*broaden*; Mikulincer & Shaver, 2007).

Thus, individuals with high levels of mentalization typically show considerable resilience in the face of stressful conditions, and are often able to gain a surprisingly beneficial perspective on their lives as a result of adversity (Fonagy et al., 1994). Moreover, they show good capacities for both relationship-recruiting—i.e., the capacity to become attached to caring and helpful others (Hauser et al., 2006)—and effective coregulation of stress and adversity (Luyten et al., 2009). These individuals typically also have a good capacity to explore both the external world and their own internal world. This is often manifested in marked creativity; the ability to symbolize; the ability to shift perspectives on their lives and that the lives of others; interest in dreams, fantasies, art and music; and general interest in the internal worlds of other people.

Mindfulness of other minds is one of the best indicators for high levels of mentalization, and is associated with a sense of internal freedom to explore thoughts, feelings, desires, and experiences. Mature mentalizers have the inner security to explore and verbalize even difficult memories and experiences, and are clearly interested in doing so (2008). This security in mental exploration, which may be driven by both positive and negative experiences, also promotes the capacity to call for and accept help (Grossman et al., 1999) But this is not the story for people with BPD: They use markedly different attachment strategies that have devastating effects on their ability to reflect on themselves and the motives of others. This, in turn, seriously and negatively impacts their interactions and relationships.

### Hyperactivation Strategies

To manage their internal states and interactions with others, people with BPD tend to use hyperactivation strategies (although some may resort to deactivation procedures, as described in the following). Attachment hyperactivation strategies (Mikulincer & Shaver, 2007) are displayed by the anxious patient with BPD who attaches to others easily and quickly. This often results in disappointment for two reasons: first, attachment hyperactivation causes individuals to form inappropriately intense attachments to others; second, it inhibits neural systems associated with judging the trustworthiness of others (Allen et al., 2008; Fonagy & Bateman, 2008. Thus, BPD patients rapidly idealize their treatment and therapist, become overtrusting, and show tendencies to overstep normal social scripts including therapy/clinical scripts.

When their needs are not met, however, BPD patients can quickly reverse their strategies. As a result, these patients become dismissive, hostile, and critical. They also show an increased time to recovery of mentalization. In response to active probing or challenging automatic assumptions during assessment, clinicians should take loss of mentalization as a clear warning about the sensitivity of the patient's attachment system. With such patients, it is dangerous to offer treatment in an environment overstimulating of attachment (for example, an in-patient unit). Similarly, a therapy that intensifies the patient–therapist relationship too early should not be recommended. In MBT, as a matter of principle, the intensity of the patient–therapist relationship is only intensified later in treatment when the patient is able to maintain mentalizing during more intimate interpersonal interactions.

### Deactivation Strategies

By contrast, individuals who primarily use attachment deactivation strategies, such as emotional distance, are able to keep mentalization longer on-line. In the face of interpersonal stress, they emotionally distance themselves. Under increasing levels of stress, these deactivating strategies tend to fail, leading to a strong reactivation of feelings of insecurity, heightened activation of negative self-representations, and increased levels of internal distress (Mikulincer, Dolev, & Shaver, 2004).

Research has shown that individuals using deactivating strategies may show considerable biological stress indications (such as increases in blood pressure). At the same time, however, these individuals not only appear to be calm; they also report that subjectively they feel nondistressed (Dozier & Kobak, 1992; Luyten et al., 2009). The following observations potentially indicate a dissociation between subjective and biological distress: Individuals appear too calm for the situation (e.g., talking about a history of emotional neglect without showing any signs of discomfort); they cannot provide examples, such as specific attachment experiences, illustrating general statements; or they first appear calm, but then suddenly become extremely uncomfortable (e.g., they start sweating, or suddenly start feeling dizzy). In addition, these individuals often attribute sudden changes in their distress levels not to the topic under discussion, but to external circumstances (e.g., that they have not eaten enough that day and therefore feel dizzy). Under these circumstances, the clinician needs to be aware that what you see is not necessarily what you get. The patient may appear to be able to mentalize but is, in fact, using rational and intellectual processes devoid of affect—processes that are more akin to pretend-mode functioning.

### Mixed Strategies

Individuals with disorganized attachment may show both marked deficits in mentalization and a tendency for hypermentalization (Bateman & Fonagy, 2004a). They use deactivating strategies when hyperactivating strategies fail (or vice versa). This often results in marked oscillations in mentalizing. As a result of an overreliance on modes of social cognition that antedate full mentalizing, hyperactivating strategies lead to a loss of mentalization and thus to failures in understanding the mental states of self and others (Bateman & Fonagy, 2006). Attachment deactivating strategies, however, are typically associated with minimizing and avoiding affective contents. As a result, an individual employing such strategies has a tendency to hypermentalize through continuing, but unsuccessful, attempts to understand their own and others’ mental states. This pattern is reflected in the empirical observation of both fearful-avoidant and preoccupied attachment styles (Choi-Kain et al., 2009).

The assessing clinician will be able to map these strategies by taking a detailed account of the patient's intimate relationships and carefully exploring the patient's behaviors (such as suicide attempts and self harm). For example, patients who frequently use hyperactivation strategies may complain that their relationships start well, and that they rapidly fall in love, but then they find themselves betrayed, cheated, deceived, and neglected. Not surprisingly, this leads to a sudden breakdown in their relationships. If the assessor probes, the patient will show limited reflection about their own role in the problem, although he or she may say that it must be his or her own fault because it keeps happening.

The patient who uses more deactivating strategies may lead a somewhat isolated life and limit their interactions with others, rarely forming intimate attachments. They may spend considerable lengths of time engaged in solitary pursuits. At interview, challenges and detailed exploration will elicit limited emotional response, and the interviewer may even become bored. Most patients engage in a mix of strategies, and the assessment process should identify the circumstances likely to trigger one or other of the strategies. This will inform the assessor about areas of sensitivity, which in turn allows the patient and therapist to identify areas of treatment that might become problematic.

## MENTALIZATION-BASED TREATMENT

It should be apparent from this discussion about attachment and BPD that the focus of treatment needs to be on stabilizing the sense of self, sustaining mentalizing within the interpersonal context of therapy, and helping the patient maintain an optimal level of arousal during interactions with others. Problems with mentalizing in patients with BPD can be suumarized as follows:

1. As a result of hypersensitivity of the attachment process, patients with BPD are vulnerable to losses of mentalizing in the context of attachment relationships.
2. Loss of mentalizing leads to prementalistic modes of functioning—psychic equivalence (concrete), pretend mode (dissociated), and teleological (action and outcomes oriented) mode of subjective experience.
3. In these states of mind, experiences are either too real or meaningless, and the patient's understanding of motives is solely in terms of the physical world—i.e., things have to happen or be done to be meaningful.
4. These distortions of subjectivity are commonly accompanied by intense psychic pain that is hard for those not sharing the patient's experience to fully appreciate.

To address these key points, we have defined some core techniques that are to be used in the context of group and individual therapy. We have labeled these techniques MBT (A. W. Bateman & Fonagy, 2004). This treatment can be implemented by generic mental health professionals with experience of working with personality disorder; only moderate levels of additional training are required.

The initial task in MBT is to stabilize emotional expression. Without improved control of affect, there can be no serious consideration of internal representations. Although the converse is true—without stable internal representations there can be no robust control of affects— identification and expression of affect are targeted first because they represent an immediate threat to continuity of therapy (as well as potentially to the patient's life). Uncontrolled affect leads to impulsivity. Only once affect and impulsivity are under control is it possible to focus on internal representations and to strengthen the patient's sense of self. In order to implement the treatment, the stucture and context of treatment first need to be defined and organised.

## STRUCTURE OF MBT

A range of therapies are effective in the treatment of BPD (Davidson et al., 2006; Gieson-Bloo et al., 2006; Linehan et al., 2006; Clarkin, Levy, Lenzenweger, & Kernberg, 2007; A. Bateman & Fonagy, 2009; McMain et al., 2009). Although they are diverse in focus, treatments that have been shown to be moderately effective for BPD have certain common features (A. Bateman & Fonagy, 2000). They tend to:

1. Be well-structured;
2. Devote considerable effort to enhancing compliance;
3. Have a clear focus (whether on a problem behavior, such as self-harm, or on an aspect of interpersonal relationship patterns);
4. Be theoretically highly coherent to both therapist and patient, sometimes deliberately omitting information incompatible with the theory;
5. Be relatively long-term;
6. Encourage a powerful attachment relationship between therapist and patient, enabling the therapist to adopt a relatively active rather than a passive stance; and
7. Be well integrated with other services available to the patient.

Although some of these features may seem to characterize a successful research study instead of a successful therapy, the manner in which clinical treatment protocols are constructed and delivered is probably distinctly important in the treatment of BPD. Part of the benefit that borderline personality disordered individuals derive from treatment comes through their experience of being involved in a carefully considered, well-structured, and coherent interpersonal endeavor. What may be helpful are the internalization of a thoughtfully developed structure, the understanding of the interrelationship of different reliably identifiable components, the causal interdependence of specific ideas and actions, the constructive interactions of professionals, and, above all, the experience of being the subject of reliable, coherent, and rational thinking. It may be argued on empirical grounds that BPD patients have been deprived of exactly these sorts of social and personal experiences during their early development and frequently throughout their life. The provision of such experiences in treatment probably correlates with the level of seriousness and the degree of commitment with which teams of professionals approach the problem of caring for this group of patients. The organization and delivery of MBT takes into account all these aspects of treatment.

MBT is organized around an 18-month treatment period commencing with an assessment procedure and introductory sessions. This is followed by weekly individual and group therapy accompanied by crisis planning and integrated psychiatric care (A. Bateman & Fonagy, 2004b, 2006).

## ASSESSMENT

A detailed knowledge of the specific types of impairments in mentalization—and particularly the specific attachment contexts in which these impairments are manifested—may not only inform the focus of treatment; it may also inform the assessor and future group and individual therapist about the type of relationship and associated mentalizing deficits that are likely to develop in treatment. Therefore, an evaluation at the level of clinical practice of individuals’ mentalizing depends on detailing their uses of the primary attachment strategies discussed earlier.

Following the assessment of mentalizing, the patient and therapist discuss the diagnosis of BPD and start to consider the main presenting problems in terms of difficulties in mentalizing. This leads to a formulation that is a joint therapeutic task between the patient and therapist. In MBT, the purpose of the formulation is not to identify the truth about the patient's problems. Rather, the goal is to initiate the process of mentalizing. The therapist has to identify the patient's reasons for seeking treatment and place them in a historical and current context, while at the same time juxtaposing his own understanding of the patient's experience. The patient is asked to articulate his own experiences while simultaneously reflecting on the therapist's experience, as it is verbally presented to the patient by the therapist. This necessitates the therapist being more open about what is in his mind than is traditional in dynamic therapies. In return, the therapist agrees to consider his own state of mind in relation to the patient's verbalized experience without assuming that his understanding has greater validity. At best, it is an alternative perspective.

This humility with regard to understanding motives and underlying mental states begins with the initial formulation, but is also key to MBT as a whole. The emphasis placed in treatment on the therapist stance is one of MBT's basic components.

## THERAPIST STANCE

The therapist's mentalizing therapeutic stance should include:

1. Humility deriving from a sense of not-knowing;
2. Patience in taking time to identify differences in perspectives;
3. Legitimizing and accepting different perspectives;
4. Actively questioning the patient about their experience—asking for detailed descriptions of experience (*what* questions), rather than explanations (*why* questions); and
5. Careful eschewing of the need to supply understandings for things that do not make immediate sense—the therapist is instructed to say explicitly that something is unclear.

An important component of this stance is monitoring one's own misunderstandings as a therapist. Acknowledgment of misunderstanding not only models honesty and courage; by taking responsibility for misunderstandings, the therapist also tends to lower the patient's level of arousal. Furthermore, acknowledgement offers invaluable opportunities to explore how misunderstandings can arise out of mistaken assumptions about opaque mental states, and about how these misapprehensions can lead to massively aversive experiences.

In this context, it is important to be aware that the therapist, when working with a nonmentalizing patient, is constantly at risk of losing his own capacity to mentalize. Consequently, we consider therapists’ occasional enactments acceptable concomitants of the therapeutic alliance, something that simply has to be owned up to. As with other breaks in mentalizing, such incidents require that the process is rewound and the incident explored. Thus, in the collaborative patient–therapist relationship, the two partners involved have a joint responsibility to understand enactments.

## THERAPIST ATTITUDE

The attitude of the therapist is crucial. The therapist's task is to stimulate a mentalizing process and make it an essential feature of the therapeutic interaction. Thinking about oneself and others develops, in part, through a process of identification: The therapist's ability to use his mind and to demonstrate a change of mind when presented with alternative views is internalized by the patient. Gradually, the patient becomes more curious about his own and others’ minds, and is consequently better able to reappraise himself and his understandings of others.

The continual reworking of perspectives on self and others in the context of an intense attachment relationship is key to the process of change. So, too, is focusing the work on current, rather than past, experience. The therapist's task is to maintain mentalizing and/or to reinstate it in both himself and his patient while ensuring that emotional states are active and meaningful. On the one hand, excessive emotional arousal will impair the patient's mentalizing capacity and potentially lead to acting out. On the other hand, inadequate emphasis on the relationship with the patient will allow avoidance of emotional states. In turn, this limits the contexts within which the patient can function interpersonally. The addition of group therapy to individual sessions dramatically expands the contexts in which this process can take place—hence the use of both individual and group modes in MBT.

## THE NOT-KNOWING STANCE

The not-knowing aspect of the mentalizing stance is part of this general therapeutic attitude and is central to ensuring that the therapist maintains his curiosity about his patient's mental states. He must accept that both he and his patient experience things only impressionistically and that neither of them has primacy of knowledge about the other or about what occurs between them.

This principle is more easily articulated in theory than achieved in therapy. Both patient and therapist can behave as if they are sure about what the other is thinking or feeling. When did a therapist last say to a patient, “You must be feeling *…* ?” The use of the word *must* implied that the therapist knew what the patient was feeling even if the patient had not expressed the feeling. No doubt the motive for making the statement was to increase the therapeutic alliance through empathy. Of course, the therapist might be correct about the feeling of the patient; but, equally, he or she might be wrong. A therapist's representation of a feeling can never be the same as the patient's representation.

BPD patients will all too easily agree with the therapist's suggestion, taking on his or her mental state and entering pretend-mode functioning. This circumscribes their exploration of their own mental processes, and prevents them from discovering exactly what they do feel. In MBT, it is better to ask, “What is it that you feel about that?” Only if a patient struggles to answer should the therapist nudge the patient by saying, “If it were me, I would feel *…*,” or, “It sounds to me like you feel *…* .” Both statements are less prescriptive and are marked as abstractions arising from the therapist's experience.

Although all this might be implicit when one says to a patient, “you must feel *…*,” the patient's state of confusion and her lower mentalizing capacity will ensure that she experiences such a statement as a fact about how they feel, rather than experiencing it as a prompt to consider further their feeling. So, unwittingly, the therapist has taken over the patient's mental states rather than stimulating their independent development.

A common confusion has been that being a not-knowing therapist is equivalent to feigning ignorance. Nothing could be further from the truth. The therapist has a mind and is continually demonstrating that he can use it. He commonly has alternative perspectives from the patient and, if so, this creates an excellent opportunity for further exploration. Once the therapist has established the process associated with the not-knowing stance, the model recommends that the therapist sensitively increases the focus on the relationship between patient and therapist. The task here is to stimulate the attachment process within the context of an ever-increasing intimacy, which is the area of sensitivity for people with BPD. It has already been mentioned that intimacy within interpersonal contexts more readily provokes loss of mentalizing in patients with BPD than it does in other patients. At the same time, however, it is this context in which mentalizing is most needed. So, the MBT therapist works *in vivo*, replicating the conditions in which a patient might lose mentalizing, while at the same time helping him maintain it.

## BASIC INTERVENTIONS

We have suggested that interventions are organized around a series of therapeutic steps:

1. Demonstrating empathy with the patient's current subjective state;
2. Exploration, clarification and, if appropriate, challenge;
3. Identifying affect and establishing an affect-focus; and
4. Mentalizing the relationship.

Detailed discussion of these therapeutic interventions can be found in A. Bateman and Fonagy (2006). Although the description appears prescriptive and reductionist, the interventions are described in this way for clarity and to highlight their potential both for effectively reinstating mentalizing and for causing iatrogenic harm. In MBT, the therapist follows the principle that if the patient is emotional with a resulting loss of mentalizing, then only safe interventions can be used. In this context, a safe intervention is one that decreases arousal, allowing for the best chance of reinstating mentalizing. In effect, it is an intervention at the first or second levels listed previously. These are safer simply because they are less likely to stimulate further arousal and problematic emotional states and do not require a high level of mentalizing capacity on the part of the patient. Once the patient is able to reflect to some extent on current states of mind, it is possible to consider expanding the therapeutic process using interventions at the third and fourth levels. Thus, in practice, the MBT therapist moves around the levels according to his sensitivity to the patient's arousal level and mentalizing capacity. Over time, this helps the patient achieve greater mentalization of the relationship between patient and therapist.

It has been suggested that MBT does not make use of transference (Gabbard, 2006). This is incorrect. Transference interpretation is a high-order technique not easily learned. Dynamic therapies that effectively employ it have often been criticized for being complex and difficult to implement well without extensive training. The technique may harm BPD patients if inappropriately used. MBT was developed as a research-based treatment to be quickly learned and easily implemented by generic mental health professionals. It is designed to avoid possible harmful effects of overzealous, clumsy transference interpretations delivered without the balancing statements described by expert practitioners (Fonagy & Bateman, 2006). For this reason, MBT practitioners cautiously approach the use of transference.

In our trials of MBT, therapists have been practitioners with no specialist psychotherapy training. MBT itself has minimal training and supervision demands because it uses a commonsense view of the mind, incorporates generic ideas from different models of psychotherapy, blends them into a healthy ecumenism relevant to BPD, and meets realistic service and training patterns. Three days’ basic training is provided. Supervision is offered in the workplace as practitioners see patients for treatment. Current results suggest that reasonable outcomes may be achievable within this framework of mental health services without lengthy specialist training. This supports the general utility of MBT.

Some of the success may be due to avoiding iatrogenic interventions, and so we caution practitioners first about the commonly stated aim of transference interpretation, namely to provide insight, and second about genetic aspects such as linking current experience to the past because of their potential iatrogenic effects. Many different interventions come under the guise of transference interpretation. So we have been careful to define what we mean. In our initial manual, we outlined this and in our most recent practical guide we provide detailed illustration of transference interpretation in MBT). Our use of transference concurs with the outline given in the paper by Per Hoglend (2006), except we caution against genetic transference links.

Our first step is the validation of the transference feeling. The danger of the genetic approach to the transference is that it might implicitly invalidate the patient's experience. The second step is exploration. The events generating the transference feelings must be identified and the behaviors tied to these thoughts or feelings need to be made explicit, sometimes in painful detail. The third step is accepting enactment on the part of the therapist. It is likely that most experiences of the patient will be based on reality, even if the connection to it is very limited. This means that the therapist has been drawn into the transference and acted in some way consistent with the patient's perception of her. It may be easy to attribute this to the patient but this would be completely unhelpful as it will only serve to invalidate the patient's experience totally. The therapist should explicitly acknowledge even partial enactments of the transference as inexplicable voluntary actions that she or he accepts agency for, rather than identifying them as a distortion of the patient. Such therapist actions will help demonstrate to the patient that one can accept agency for involuntary acts without invalidating the attitudes one is trying to convey. Only then can distortions be explored. Step four is a collaboration aiming to arrive at an alternative perspective; in MBT this is an expansion of the understanding of the events and experience under scrutiny. As with any other forms of mentalizing, this is a collaborative effort, and the therapist must imagine sitting next to the patient, not opposite. They sit side-by-side looking at each other's thoughts and feelings, both adopting the inquisitive stance where possible. The fifth step is when the therapist summarizes the alternative perspective. The final step is the careful monitoring of both the patient's and one's own reaction.

We suggest taking these steps in sequence and we commonly talk about mentalizing the relationship, rather than mentalizing the transference to distinguish the process from transference interpretation, which is commonly viewed as a technique to provide insight. *Mentalizing the relationship* is a shorthand term for encouraging patients to think about the relationship they are in at the current moment (the therapist relationship) with the aim of focussing the patient's attention on another mind, that of the therapist, and to help the patient contrast her own perception of herself with how she is perceived by another, by the therapist or indeed by members of a therapeutic group.

## CASE EXAMPLE

A 26-year-old unemployed woman living in social housing was referred to our service because of persistent suicide attempts, self-harm, poor social function, and chaotic personal relationships. She had two children who were in care of social services, and she stated that she wanted them back At interview she was sullen, sat hunched forward throughout with a baseball cap pulled low so that her face was covered, and exhibited blood-stained bandages on both wrists. She gave an account of having been an unwanted child of a drug-addicted father and a mother who had multiple partners. She had been abused by one of her mother's boyfriends. Her education was rudimentary, but she had learned to read and write. At initial psychiatric assessment, she was considered to be of average intelligence. One constant figure in her life was her maternal grandmother, who brought her up from the age of 5 to 16 years. She then moved in with a man older than her and became pregnant six months later with her first child. Her second child was conceived during a drug binge with acquaintances, and the father was not known to her.

The initial interview confirmed that she had BPD. She was offered group plus individual MBT, both on a weekly basis. She stated that she did not want a group. The MBT therapist discussed with her the anxieties that she had about attending a group and was empathic about the difficulties that she might face because she hated being looked at by people. The therapist asked her if that was why she kept her cap on in the interview. She said that she didn't really want anyone to see her. The therapist said that the treatment team would take this into account and alert the group therapist to her panic about starting in the group. Finally, the assessor said that the program required attendance at both components of the program, and the patient reluctantly agreed. Illustrative elements of the 18-month treatment are now discussed from the perspective of the individual therapy.

### Intake

The patient started a 12-week, fixed-term psycho-educational group where she took part in exercises covering topics related to personality disorder, mentalizing, attachment, self-harm, emotions, and group and individual therapy. After this preparatory phase, the patient and original assessor discussed the treatment program and together they worked out and agreed on a crisis plan. This plan is a reference point for both the patient and therapist and is to be used if the patient feels unable to cope and is at increasing risk of becoming self-destructive. The MBT practitioner may be contacted by the patient as part of the crisis plan, but only if the patient has first attempted to reduce panic by other means. The plan forms the foundation of continuing therapy.

### Session

The patient began the session.

**Table:** 

Patient:	I have nothing to say today. I am blank.
Therapist:	What is it like to be in a session sitting with me but having nothing to say and nothing in your mind?
Patient:	Hmm. Uncomfortable.
Therapist:	I can see that. I suppose that there is a bit of pressure to say something to reduce how uncomfortable you feel.
Patient:	I have to talk; otherwise it will be worse with you looking at me.
Therapist:	Is there anything particular about today that is making it problematic, or is there anything I can do to help this?

The session continued with the therapist trying to stimulate a dialogue while simultaneously attempting to avoid guessing what was going on in the patient's mind or telling the patient why she has nothing to say. MBT practitioners should start with an empathic intervention but address two components of empathy. First, there is the affective state; and second, the therapist needs to address the consequence for the patient of her affective state in the context she is in. This therapist does so here. He identifies both the patient's blankness and discomfort and the effect that this has in the current moment of therapy. The therapist also places himself into the frame with the patient by asking if there is anything that he can do to help. After this comment, the therapist was silent. In general terms, the MBT therapist is active as the therapy is not seeking to identify unconscious concerns and process, so free association, for example, is not a technique that is used. The patient broke the silence.

**Table:** 

Patient:	I feel uncomfortable about things at the moment and do not want to go to the group any more. I find Jenny (another patient) impossible. She talks all the time and the therapist does not ask her to keep quiet. At the last group I walked out because she did not stop talking, so it was a waste of time me being there. No one else had a chance.
Therapist:	Can you say what it was you were feeling at the time?
Patient:	The therapist in the group should be doing something to help us and should take charge. I felt useless as I can't do anything. He gets involved with Jenny and ignores us.
Therapist:	Sounds like we have to think about how to get the therapist to do something.
Patient:	I am not going to the group anymore.
Therapist:	Before we get there, I think we should discuss what this therapist is up to.

Here the therapist moves alongside the patient to explore her criticism of the group therapist. In MBT, the individual therapist and group therapist work jointly. Problems in the group are discussed in the individual therapy and any difficulties in the individual work are attended to in the group. Initially the therapist does not remain neutral about where the problem lies—with the therapist, the group, or the patient—but errs on the side of the patient by implying that there is a problem with the group or therapist, rather than with the patient herself. In MBT, the therapist is expected, in the first instance, to see things from the patient perspective, to validate how they perceive the problem. Patients with BPD are very externally focussed, for example listening for nuance in tone of voice, and exquisitely sensitive to suggestions that they, themselves, are the problem. They easily feel that their perceptions are invalid and that they are blamed for everything. This alienates them from the interaction and disrupts mentalizing.

**Table:** 

Patient:	I don't think she really knows what she is doing. I am not even sure that she notices us. The last group was nearly all about Jenny and of no relevance to most of us. Other patients think so too. It is not just me. We talked about it after the group last week. No one takes any notice of us. I don't want to talk about it anymore.
Therapist:	What makes you say that so suddenly?
Patient:	Nothing really. It is not worth talking about so I don't want to talk anymore about it. The real problem for me is that my car has broken down and I cannot afford to have it repaired. So I feel frustrated.

This sudden change in subject and the emphatic statement by the patient that she was not going to talk about the group further led to a palpable uncertainty in the session. The therapist was unclear whether to push more about the group or to leave the subject alone. It is in this situation in MBT that the therapist considers trying to identify the affect focus of the session in the moment: the immediate affective component of the session that is shared between patient and therapist. Initially, identification of the affect in the session is usually voiced, that is marked, as being the creation of the mind of the therapist rather than stating something about the mind of the patient.

**Table:** 

Therapist:	Before we go to the problem with the car, I am left wondering if I should push you a bit more to talk about the problem with the group therapist or like you, feel it would be best left alone. May be neither of us knows whether to talk more or to stop.
Patient:	Well, I want to stop. You might not believe me and just be thinking that I am being too sensitive again and should speak out and definitely go back to the group.
Therapist:	I suppose that is true, as I don't know what has been going on, but to me you seem quite clear about what has happened, rather than being sensitive. I am not quite sure what it is about it though that means that you won't go back to the group.
Patient:	Because I can't say anything.
Therapist:	What makes you so sure about that?

The therapist here tries to identify with the patient's unspoken concern as to whether her perception of events is valid. But the therapist accepts the patient's perspective and moves the problem to the patient's dilemma of not being able to say anything and so deciding it is better to leave the group. When the patient suggests that she cannot say anything in the group, this therapist focuses on how that statement is held, rather than the statement itself. This is typical of MBT; it is how ideas are held, for example the absence of doubt, the quality of the discourse, that indicates whether the patient is mentalizing or not. The therapist could equally have addressed the certainty with which the patient held her perception of the inadequacy of the therapist. The therapist's primary task is to help the patient to mentalize again. It is not to find some absolute truth.

**Table:** 

Patient:	I just know that I can't. I don't want to say more about it now

The use of *just* commonly indicates continuation of nonmentalizing and closing down of mental processes, so the therapist begins to explore this in more detail. It transpires that the patient felt saying something critical of the therapist might draw too much attention to herself, and she would feel “looked at” and people would gang up against her. At this point, the therapist begins to mentalize the relationship.

**Table:** 

Therapist:	You know that reminds me that you didn't want to say anything at the beginning of the session either and you don't want to say more now. Can we go back slightly to see what happened now to make you feel that you don't want to say more? It occurs to me that I was thinking and looking at you without saying anything.
Patient:	When you don't speak it makes me nervous.

Here the therapist rewinds the session to identify the process that might have turned off the patient's mentalizing. He suggests that his own behavior might be the reason why the patient is unable to continue exploring the problem. Only if this is addressed is the patient likely to be able re-instate mentalizing.

**Table:** 

Therapist:	Yes. I am sorry about that, as I know how difficult that feeling is for you. You hate being looked at by people. Is that your feeling at the time when you can't say anything?
Patient:	I feel uncomfortable about saying things to you because you have to take the side of the group therapist. You are bound to support her.
Therapist:	I am not quite sure that is correct, because if she is not supporting you and leaving the other patient to dominate the group to your detriment, then it is important that we all address it.

In this section, the therapist tries to validate the patient's anxiety while also questioning her assumptions.

Therapist: Is there something else in this sense that you cannot talk to me and hide yourself a bit [the patient had pulled her cap over her head more firmly]. Can you tell me how you feel now?

**Table:** 

Patient:	No. Just ashamed.
Therapist:	There is that “just” word again. In what way ashamed?
Patient:	Exposed and wanting to cover myself up.
Therapist:	So tell me about what it is you are covering up.
Patient:	Everything about me. If I tell you what is happening, then you will go against me and take the side of the group therapist. Then I will be on my own and all because I talked about something I shouldn't have done. I think I will go now.
Therapist:	I don't think you should leave. If you can stay we can see what it is that makes you so sensitive to your fear that I might not support you in your experience in the group and how you become so ashamed.
Patient:	Will you tell him what I have said?
Therapist:	I think I should because it is important that he knows about your distress in the group, and it is equally important that you do so as well. What would you like me to do?
Patient: Not sure.

The patient and therapist carry on exploring this aspect of their relationship. They also address the patient's anxieties about not being believed or not being supported in her perception of the group therapist and her concerns that the individual and group therapists would organize themselves against her in some way. The patient became more receptive to this process after the therapist's interventions and reassurance. This is the process of mentalizing the relationship. There is no specific genetic interpretation. The therapist is more concerned with paying attention to the relationship and how it affects the patient. It might be possible to relate the current process to a similar process in the past, for example with this patient her attempts to talk to her mother about the sexual abuse from the boyfriend had led to her mother disbelieving her and becoming closer to this boyfriend. In MBT, however, this is not the aim. The therapist is more concerned with generating a reflective process than stimulating insight, but self-understanding may come as an epiphenomenon of this process.

## OVERVIEW OF STUDIES

Evidence base for any therapy comes from two distinct sources. There are outcome studies that support the value of a treatment, but these cannot stand on their own. The mechanism of pathology and cure postulated within a theoretical frame must also be validated. David and Montgomery (2011) proposed that to assess the extent of evidence base for each of the psychotherapies they should be first classified into nine graded categories, ranging from evidence-based psychotherapies at one end to psychotherapies with problematic theories and ineffective interventions at the other. The evidence base of therapies is defined by two factors: (a) theory (mechanisms of psychological change) and (b) therapeutic package derived from that theory, and each factor is organized by three levels: (a) empirically well supported; (b) equivocal data and (c) strong contradictory evidence. There should also be a clear relationship between a guiding theoretical base and the empirical data collected.

The outcome of MBT is supported by only two randomized controlled trials, both carried out by the developers of the treatment. It has been shown that a manualized mentalization focus in the context of day hospital care serves to reduce impulsive symptoms of BPD including suicidality and self-harm, as well as depression (A. W. Bateman & Fonagy, 1999). In this initial study, forty-four patients were randomized either to the specialist mentalization treatment or to treatment as usual. Gains were made by both groups, but the improvements in the specialist treatment were significantly better. The improvements observed at 18 months and the differences between the groups were maintained at 36 months (A. W. Bateman & Fonagy, 2001) An 8-year follow-up surprisingly revealed that many of the benefits were still evident in terms of suicide attempts and hospitalization, and social functioning in terms of employment and use of services also favoured the MBT group (A. Bateman & Fonagy, 2008). Health costs of the treatment were recovered in the course of 36 months and a case for cost-offset could be made (A. W. Bateman & Fonagy, 2003). The sample of the day hospital treatment trial was small and the treatment included multiple components in addition to the manualized group and individual psychotherapy.

A larger trial comparing outpatient therapy with structured clinical management showed more compelling outcomes. Patients with BPD who were consecutive referrals were randomly allocated (minimization for age, gender, antisocial personality disorder) to intensive outpatient and structured clinical management (SCM) groups. Both groups received individual therapy (50 mins) and group therapy (1.5 hrs) sessions weekly for eighteen months delivered according to a detailed manuals. Both treatments offered support and structure, challenge of self-destructive behavior patterns, protocolized medication review and crisis management. Althoughthe MBT included enhancement to basic mentalizing, interpretive mentalizing interventions, and mentalizing the therapeutic relationship (see mentalizing the transference), SCM focused on advocacy, social support, and support for work activity, as well as interpersonal and individual problem solving. The study was unusual in coopting or recruiting naive therapists for the trial and randomly assigning them to a three-day training in MBT or SCM with continued supervision. Eleven therapists who had an interest in MBT with minimum of two years’ experience of treating patients in general psychiatric services following their generic training and a minimum of one year's experience treating patients with personality disorder were randomized to one or other arm. They did not differ in their years of psychiatric experience or professional qualification. Patients were assessed at admission, 6 months, 12 months, 18 months. One hundred and sixty-eight patients were screened and 134 were randomized; 71 patients allocated to MBT, and 63 patients allocated to SCM. Intent to treat analysis included all patients who started the trial. Primary outcome was the proportion of each group without severe parasuicidal behavior as indicated by (a) suicide attempt; (b) life-threatening self-harm; and (c) hospital admission formal research confirmed records. Secondary outcomes (assessed at baseline and at 6-monthly intervals until the end of treatment at 18 months) included independently rated Global Assessment of Functioning scores at beginning and end of treatments and self-reported psychiatric symptoms and social and interpersonal function using well-standardised measures. The percentage of patients who experienced a clinical episode (attempted suicide, self-harm, or hospitalization) was significantly fewer following MBT than SCM (23% vs. 57%). The secondary outcomes also favored the MBT group. It is not yet known if these gains and differences are maintained at follow-up. An observational study replicating the results was successful (Bales et al., in press). Further, independent replications are urgently needed.

In MBT, the evidence base regarding the mechanism of change is relatively sparse. Although there is strong evidence supporting the mentalization focus of the therapy for patients with BPD, there is almost no evidence that a change in mentalization is brought about by the treatment. The Cassel step-down treatment study followed 297 patients in personality disorder services. They were recruited through the Cassel Residential inpatient program (*n* = 120), the Cassel Community stepdown/outpatient programme (*n* = 113), and a management as usual group (MAU) recruited through the Devon Personality Disorder services (*n* = 64; Chiesa & Fonagy, 2007; Chiesa et al., 2011). As the model would predict, the MAU treatment did less well, but the inpatient service had poor outcome relative to the community treatment. The authors attributed this to the hyperactivation of attachment in the inpatient setting. Treatment input and staff resources were higher for the inpatient service but comparable the outpatient and MAU programs. Mentalizing, as measured by blind-rated adult attachment interview narratives, was found to be improved in the community-based program even though that was not focus of the intervention. Similar observations were reported by Levy and colleagues (Levy et al., 2006) following transference focused psychotherapy. Patients in TFP improved across a larger number of outcome criteria than those in supportive psychotherapy or DBT and only those in TFP changed in terms of their reflective function and attachment classification, suggesting that other types of interventions may also improve mentalizing.

Although empirical evidence for mentalization being the effective mechanism of change is equivocal, there is one facet of the evidence base that bears out with some force our emphasis on mentalization. As we have seen many therapies are effective with BPD and the pathway to change may indeed be via a shift in mentalizing capacity. Effective psychotherapies for BPD all have mentalizing elements. All therapies inquire into patients’ mental states, whether one calls it behavioral analysis, clarification of schemata, confrontation or interpretation. One aspect of their therapeutic action then may be strengthening the second order representations of mental states. Interestingly all the effective treatments are structured so that they provide encouragement for increased activity, proactivity and self-agency for the patients. For example, MBT eschews an expert stance, and encourages shared process of discovery captured in suggesting to therapist that they sit side-by-side with their patients. The collaborative stance of CBT is, of course, a very similar process, as is the coconstruction envisioned by cognitive analytic therapy. The attention to current mental processes is shared with TFP. All these strategies serve to enhance intentionality, underscoring for the patient how mental states drives action and their experiences are real, can be understood by another person and serve to make their actions comprehensible.

Extensive efforts are made by all therapies to maintain the patient's engagement in treatment (by validation always in conjunction with an emphasis on the need to address therapy interfering behaviours). This may be expected to generate in patients both a sense of acceptance and a sense of being recognized, or being thought about, laying the groundwork for maintaining mentalization when it would otherwise be lost. Increased cognitive coherence in relation to subjective experience is created in the early phase of all treatments by the inclusion of a model of pathology that is carefully explained to the patient. Effective therapies also have in common an active therapist stance that invariably includes an explicit intent to validate and demonstrate empathy and generate a strong attachment relationship to create a foundation of alliance. Our model suggests that the establishment of epistemic trust is essential to effective treatment. Children, and indeed adults, are probably biologically programmed to learn about their subjective worlds only from adults whose attitude they consider benign (Fonagy et al., 2007). The centrality of the therapeutic alliance to psychotherapy of all modalities is probably vested in this rather unremarkable evolutionarily preserved mechanism for cultural transmission (Gergely & Unoka, 2008).

Intriguingly, all therapies for BPD focus on emotion processing, particularly on creating robust connections between acts and feeling. For example, feelings of emptiness and confusion may be a background to self-harm, feeling rejected can be a segue to taking an overdose, feeling isolated and abandoned leading to bulimic overeating. In such cases, affect is unmentalized, poorly linked to context, experienced in the raw as one might describe it. By focusing on affect, the therapist helps to restore a cognitive representation of emotion. Affect can be overwhelming not just for the patient, but often for the therapist. It is not surprising then that all effective treatments provide a structure through their manual, which enables support for the therapist dealing with this nonmentalizing context. Further support in such contexts is, of course, offered by the supervision that is part of all evidence-based therapies. The intention behind supervision is to identify deviation from structure and provide support for adherence to various protocols, but such oversight also serves to prevent the therapist going along with nonmentalizing teleological sequences of actions. Finally, support for dealing with nonmentalizing can also be gained through the intense commitment to the particular modality that the therapist supports. Although we implicitly advocate plurality, we recognize that treatments will only be successful if therapists feel and show intense commitment to their modality. Anything less would lead to vulnerability when confronted with nonmentalizing.

## CONCLUSION

We see MBT as a collection of specific techniques for encouraging mentalization in individuals whose genetic vulnerability and early environmental experiences have made them vulnerable to losing mentalization when confronted with severe stress. The stressful experiences in the context of attachment relationships can hyperactivate the attachment system and lead to the loss of mentalization. We find that these techniques help engender a therapeutic attitude and relational context within which patients are able to recover mentalization even when challenged by social pressures. We would not claim that the techniques are unique in achieving this result or indeed that they are the most effective possible techniques for doing so. Nevertheless, we commend them as a simple but effective set of adaptations of a traditional psychoanalytic frame of reference, albeit one which moves the centre of therapeutic gravity from insight and reflection to the recovery of mentalization and social cognition.

## Notes

Anthony Bateman

Halliwick Psychological Therapies Service

St Ann's Hospital

St Ann's Road

London, N15 3TH, UK

anthony.bateman@ucl.ac.uk
